# The effect of oral probiotics in the last trimester on the human milk and infant gut microbiotas at six months postpartum: A randomized controlled trial

**DOI:** 10.1016/j.heliyon.2024.e37157

**Published:** 2024-08-30

**Authors:** Guangyu Ma, Yimi Li, Kian Deng Tye, Ting Huang, Xiaomei Tang, Huijuan Luo, Dongju Wang, Juan Zhou, Zhe Li, Xiaomin Xiao

**Affiliations:** a Department of Obstetrics and Gynecology, The First Affiliated Hospital of Jinan University, Guangzhou, China; b Department of Obstetrics and Gynecology, The Third Affiliated Hospital of Sun Yat-Sen University, Guangzhou, China; c Department of Obstetrics and Gynecology, Dangyang People's Hospital, Dangyang, China; d Department of Obstetrics and Gynecology, The Fifth Affiliated Hospital of Guangzhou Medical University, Guangzhou, China

**Keywords:** Gut microbiota, Infants, Milk microbiota, Pregnant women, Probiotic supplementation

## Abstract

**Objective:**

The main aim of this study was to evaluate the effect of oral probiotics on the human milk microbiota and determine whether that influenced infant microbiota development.

**Methods:**

A total of 27 pregnant women were recruited; 14 were assigned to the probiotic group, and the rest were assigned to the control group. Their infants were likewise assigned to the probiotic group or the control group. Pregnant women in the probiotic group received probiotic supplementation from 32 weeks of gestation until delivery. Human milk samples and infant fecal samples were collected at 6 months after delivery, and 16S rRNA sequencing was used to analyze the composition of the human milk and infant gut microbiota (NCT06241222).

**Results:**

In the control group, bacterial microbiota were detected in 8 out of 13 milk samples, whereas in the probiotic group, only 6 out of 14 milk samples contained bacterial microbiota. We examined the composition of the human milk and infant gut microbiota in both the control and probiotic groups. Spearman correlation analysis revealed that various genera in human milk were correlated with the infant gut microbiota. The Linear discriminant analysis effect size (LEfSe) showed that 6 bacteria in the human milk microbiota in the control group were significantly more abundant than those in the probiotic group. Nine bacteria were significantly more abundant in the human milk microbiota in the probiotic group than the control group. According to the LEfSe results, 11 bacteria in the infant gut microbiota in the control group were significantly more abundant than those in the probiotic group. Fourteen bacteria were significantly more abundant in the infant gut microbiota in the probiotic group than in the control group.

**Conclusion:**

The infant gut microbiota at 6 months has a complicated relationship with the maternal human milk microbiota. Oral probiotic supplementation can change the composition of the human milk microbiota and the infant gut microbiota.

## 1 Introduction

Human milk is a source of nutrients for neonates. It is an important factor affecting the early development of the gut microbiota in offspring and plays an important role in infant growth and immune development. Breastfeeding has been associated with a reduced incidence of neonatal respiratory and gastrointestinal infections and may also decrease the risk of obesity, asthma, Crohn's disease, ulcerative colitis, and diabetes, among other conditions [[1], [2], [3], [4]]. In addition, exclusive or partial breastfeeding can promote infant cognitive development [[5], [6], [7]]. The microbiota in human milk plays an important role in stimulating the immune system in newborns, which is conducive to the development of the intestinal microbiota with immunomodulatory activity in newborns [8].

The maternal gut microbiota may be the source of the milk microbiota. Some scholars have proposed the concept of “the bacterial entero-mammary pathway”. During pregnancy, maternal intestinal permeability increases, leading to gut bacterial translocation to the lamina propria and Peyer's plaques along with mucosal dendritic cells. Then, gut bacteria can migrate from Peyer's plaques to mesenteric lymph nodes and enter the breast via lymph vessels and peripheral blood [[9], [10], [11], [12]]. Human peripheral blood mononuclear cells (PBMCs) and milk have been found to contain bacteria and their genetic material. These bacteria are phagocytosed by PBMCs and transferred to the mammary gland intact. Currently, it is widely believed that human milk is nonsterile.

Probiotics are defined as live microorganisms that are beneficial to human health [[13], [14], [15]]. Previous work has shown that probiotic supplementation during pregnancy can reduce maternal depression or anxiety and reduce the risk of gestational obesity and gestational diabetes [[16], [17], [18]]. Probiotics can also reduce the incidence of allergic diseases such as eczema or special dermatitis during pregnancy [19,20]. The gut microbiota during pregnancy can affect the composition of the early lactation microbiota in the first two weeks of the postpartum period [21]. Human milk is the best source of nutrition for infants, and the World Health Organization advocates exclusive breastfeeding until six months of age [22]. The aim of this study was to observe the effect of oral probiotics administered during the last trimester on microbiota in human milk and infant gut. We also wanted to determine the relationship between human milk and the infant gut microbiota.

## 2 Materials and methods

### 2.1 Participants

All methods were performed in accordance with relevant guidelines and regulations for clinical trials (NCT06241222). Normal singleton pregnant women who had undergone a systematic birth examination at the First Affiliated Hospital of Jinan University and who were scheduled to give birth at our hospital were selected. The inclusion criteria were as follows: Chinese nationality, singleton pregnancy, pre-pregnancy BMI of 18.5–24 kg/m^2^, first normal pregnancy, infants exclusively breastfed for 6 months, and infants delivered vaginally. The exclusion criteria were women of an pregnant women over 35 years old, with a history of gastrointestinal tract issues, with a history of vaginal infection before pregnancy, and with hypertension, diabetes, immune diseases, gestational hypertension, gestational diabetes, or other pregnancy complications and those taking probiotics or antibiotics during lactation. Informed consent was obtained from every participant before 32 weeks of pregnancy.

A total of 27 pregnant women meeting the inclusion criteria were recruited and randomly assigned to either the probiotic group (14 participants) or the control group (13 participants). Pregnant women in the probiotic group were advised to consume live combined *Bifidobacterium*, *Lactobacillus*, and *Streptococcus* tablets twice a day after 32 weeks of pregnancy (Inner Mongolia Shuangqi Pharmaceutical, 0.5 g/tablet; each tablet contained live bacteria of *Bifidobacterium longum* of no less than 0.5 × 10^7^ CFU and live *Lactobacillus bulgaricus* and *Streptococcus thermophilus* of no less than 0.5 × 10^6^ CFU), hereinafter referred to as triple-bacteria tablets, until childbirth.

Postpartum human milk and infant fecal specimens were collected for a period of 6 months. In the control group, due to personal reasons, one infant fecal specimen was not obtained, resulting in a total collection of 13 human milk samples and 12 infant fecal samples. In the probiotic group, two infant fecal specimens were not collected, while 14 human milk samples and 12 infant fecal samples were successfully obtained. The human milk samples from the control group were designated CM, and the infant samples were designated CI; however, in the probiotic group, the human milk samples were labeled PM, and the infant samples were labeled PI. The detailed sample collection information is presented in Table 1, Table 2.

**Table 1:** Collection of human milk samples and microbial detection status.

Milk Sample Name	Samples Collected or Not	Detection of Microbial Communities
CM 1	Yes	Yes
CM 2	Yes	Yes
CM 3	Yes	Not
CM 4	Yes	Yes
CM 5	Yes	Not
CM 6	Yes	Not
CM 7	Yes	Not
CM 8	Yes	Yes
CM 9	Yes	Yes
CM 10	Yes	Yes
CM 11	Yes	Yes
CM 12	Yes	Not
CM 13	Yes	Yes
PM 1	Yes	Yes
PM 2	Yes	Yes
PM 3	Yes	Not
PM 4	Yes	Not
PM 5	Yes	Not
PM 6	Yes	Not
PM 7	Yes	Yes
PM 8	Yes	Not
PM 9	Yes	Not
PM 10	Yes	Yes
PM 11	Yes	Not
PM 12	Yes	Yes
PM 13	Yes	Not
PM 14	Yes	Yes

**Table 2:** Collection of infant fecal samples and microbial detection status.

Fecal Sample Name	Samples Collected or Not	Detection of Microbial Communities
CI 1	Yes	Yes
CI 2	Yes	Yes
CI 3	Yes	Yes
CI 4	Yes	Yes
CI 5	Yes	Yes
CI 6	Yes	Yes
CI 7	Yes	Yes
CI 8	Yes	Yes
CI 9	Yes	Yes
CI 10	Not	–
CI 11	Yes	Yes
CI 12	Yes	Yes
CI 13	Yes	Yes
PI 1	Not	–
PI 2	Yes	Yes
PI 3	Yes	Yes
PI 4	Yes	Yes
PI 5	Yes	Yes
PI 6	Yes	Yes
PI 7	Yes	Yes
PI 8	Yes	Yes
PI 9	Yes	Yes
PI 10	Not	–
PI 11	Yes	Yes
PI 12	Yes	Yes
PI 13	Yes	Yes
PI 14	Yes	Yes

### 2.2 Sample collection

When infants were 6 months old, postpartum milk was collected by two full-time members of the research group. The human milk samples were collected according to a standardized protocol [23,24]. Before collecting the samples, the nursing mothers were required to clean the nipple and areola with soap and water. A total sample volume of 6 ml was collected in a sterile tube. After collection, the samples were stored with dry ice for transportation to the laboratory immediately. Upon arrival at the laboratory, the samples were briefly thawed to allow for accurate aliquoting. Each sample was then divided equally into smaller frozen storage tubes. The divided samples were promptly refrozen and stored in a freezer at −80 °C within 4 h after collection. This process ensured that the samples were handled with minimal temperature fluctuations to preserve their integrity.

Fecal samples from the infants were collected internally in sterile and dedicated collecting boxes, thereby avoiding any contamination with foreign material. These samples were stored in a domestic freezer (−20 °C) and transferred to a laboratory freezer at −80 °C within 24 h using a cryogenic transport container to ensure they remained frozen during the transfer.

### 2.3 Detection of milk and fecal microbiota

For the fecal samples, DNA was extracted by using a Magnetic Soil and Stool DNA Kit (TianGen, China, Catalog #: DP712). For the human milk samples, DNA was extracted by using the CTAB extraction method.

16S rRNA genes of distinct regions (16SV3-V4) were amplified using specific primers (515F- 806R) with barcodes. Each PCR batch included a positive control containing a known 16S rRNA gene sequence to verify the amplification efficiency and a negative control devoid of template DNA to monitor for potential contamination or false-positive results. All PCRs were conducted with 15 μL of Phusion® High-Fidelity PCR Master Mix (New England Biolabs), 0.2 μM forward and reverse primers, and approximately 10 ng of template DNA. Thermal cycling consisted of initial denaturation at 98 °C for 1 min, followed by 30 cycles of denaturation at 98 °C for 10 s, annealing at 50 °C for 30 s, elongation at 72 °C for 30 s and extension at 72 °C for 5 min. The same volume of 1X loading buffer (containing SYB green) was mixed with the PCR products, and electrophoresis was performed on a 2 % agarose gel for detection. The PCR products were mixed in ratios with equal densities. The mixture of PCR products was subsequently purified with a Universal DNA Purification Kit (TianGen, China, Catalog #: DP214).

### 2.4 Data processing and analysis

Sequencing libraries were generated using the NEBNext® Ultra™ II FS DNA PCR-free Library Prep Kit (New England Biolabs, USA, Catalog#: E7430L) in accordance with the manufacturer's recommendations, and indices were added. The library was checked with a Qubit fluorometer and real-time PCR for quantification and a bioanalyzer for size distribution detection. The quantified libraries were pooled and sequenced on Illumina platforms according to the needed effective library concentration and data amount. Single-end reads assembly and quality control. Single-end reads were assigned to samples based on their unique barcode sequence. Each read was examined for the presence of its corresponding barcode, which was used to map the read to its respective sample. After assignment, the barcode and primer sequence were removed from each read using a sliding window approach with a minimum quality score of 20 (Phred quality score) and a minimum length of 50 bases to ensure precise truncation. This process resulted in high-quality reads that were ready for downstream analysis. Quality filtering of the raw reads was performed using Cutadapt (Martin M., 2011) (V1.9.1, http://cutadapt.readthedocs.io/en/stable/) quality control process to obtain high-quality clean reads. The following parameters were used: a quality cutoff of 30 (Phred quality score) to trim low-quality bases from the ends of the reads, a minimum length of 50 bases to retain trimmed reads, and adapter trimming using the specific adapter sequences provided by the sequencing platform. All other parameters were set to default values. The reads were compared with the reference database (Gold database, http://drive5.com/uchime/uchime_download.html) using the UCHIME algorithm (UCHIME Algorithm, http://www.drive5.com/usearch/manual/uchime_algo.html) to detect chimeric sequences, after which the chimeric sequences were removed. Then, the effective tags were obtained.

Sequence analysis was performed with Uparse software (Uparse v7.0.1001, http://drive5.com/uparse/). Sequences with ≥97 % similarity were assigned to the same OTUs. A representative sequence for each OTU was screened for further annotation. For each representative sequence, the Silva138 database (https://www.arb-silva.de/) was used based on the RDP classifier (version 2.2, http://sourceforge.net/projects/rdp-classifier/) algorithm to annotate taxonomic information.

To study the phylogenetic relationships of the different OTUs and the differences in the dominant species in the different samples (groups), multiple sequence alignment was conducted using MUSCLE software (version 3.8.31, http://www.drive5.com/muscle/). OTU abundance information was normalized using a standard sequence number corresponding to the sample with the least number of sequences. Subsequent analyses of alpha diversity and beta diversity were performed based on these output normalized data. Alpha diversity was applied to analyze the complexity of species diversity for a sample and was calculated with QIIME version 1.9.1 (http://qiime.org/scripts/split_libraries_fastq.html) [12] and displayed with R software (version 2.15.3).

### 2.5 Data normalization

The absolute abundance of ASVs was normalized using a standard sequence number corresponding to the sample with the fewest sequences. Subsequent analyses of alpha diversity and beta diversity were all performed based on the output normalized data.

### 2.6 Statistical analysis

Comparisons of alpha diversity indices between groups were performed using the Wilcoxon test with R software (version 2.15.3). The P-values were adjusted for false discovery rate (FDR) using the Benjamini–Hochberg method. Nonmetric multidimensional scaling (NMDS) was performed to obtain principal coordinates and visualize complex, multidimensional data. NMDS based on the unweighted UniFrac distance was calculated with QIIME software. NMDS analysis was performed with the WGCNA package, stat packages and ggplot2 package in R software. The significance of the differences in the sample communities was determined by linear discriminant analysis effect size (LEfSe) [25]. For the LEfSe analysis, we extracted the p-values and applied the Benjamini-Hochberg FDR correction. P < 0.05 after FDR correction was considered to indicate statistical significance. To estimate the correlation between genera, we calculated the Spearman coefcients for the top 100 genera by relative abundance. To control for multiple testing, we applied the Benjamini-Hochberg FDR correction to the p-values obtained from the Spearman correlation results. Genera with absolute Spearman correlation coefficients greater than 0.6 and FDR-adjusted P < 0.05 were considered for construction of the network. Network construction and visualization analysis were carried out using cytoscape software (Version 3.6). The Spearman correlation analysis was used to observe the correlation between human milk and the genera of infant gut microbiota, with a correlation coefficient threshold set at |r| > 0.6 and a significance level of FDR-adjusted P < 0.05.

## 3 Results

### 3.1 Status of 6-month postpartum milk and fecal microbiota library construction

In the CM, bacterial microbiota was detected in 8 of the 13 milk samples, and the detection rate for the milk microbiota was 61.5 %. In the PM, bacterial microbiota was detected in only 6 of the 14 milk samples, and the detection rate for the milk microbiota was 42.9 %. The remaining milk samples were not successfully established in the database because of insufficient bacterial content, they were not ideal for amplification and sequencing. All the fecal samples were successfully subjected to library construction for microbial analysis.

### 3.2 Constitution of the microbiota in human milk and the intestinal tract of offspring

#### 3.2.1 Human milk microbiota at 6 months

**Fig. 1:**
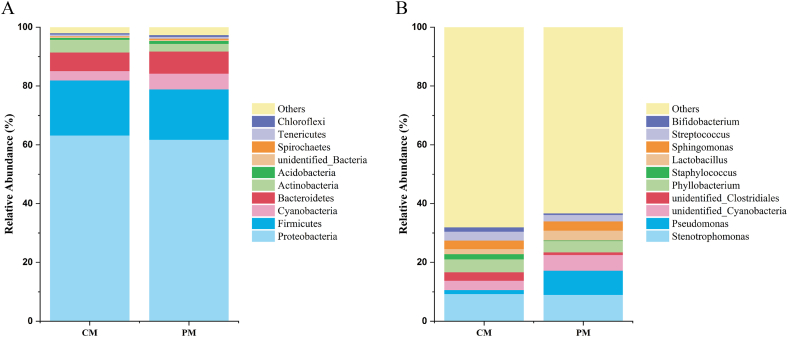
Relative abundance bar charts of the human milk microbiota species. (A) Relative abundance bar charts of the top 10 phyla in the human milk microbiota; (B) Relative abundance bar charts of the top 10 genera in the human milk microbiota. The x-axis represents sample names; the y-axis indicates the relative abundance.

*Proteobacteria* (63.2 %), *Firmicutes* (18.7 %), and *Bacteroidetes* (6.3 %) were the three most common phyla in the human milk microbiota at 6 months in the CM. Similarly, *Proteobacteria* (61.7 %), *Firmicutes* (17.1 %), and *Bacteroidetes* (7.5 %) were the three most common components in the human milk microbiota in the PM (Fig. 1A) (Table 3).

**Table 3:** Relative OTUs percentages of the top ten phyla in the human milk microbiome.

Microbiome abundance	CM (%)	PM (%)
*Proteobacteria*	63.1746	61.7189
*Firmicutes*	18.7010	17.0882
*Cyanobacteria*	3.1978	5.4046
*Bacteroidetes*	6.3067	7.4923
*Actinobacteria*	4.3088	2.6032
*Acidobacteria*	0.6727	1.0089
*unidentified_Bacteria*	0.4942	0.3797
*Spirochaetes*	0.1283	0.3827
*Tenericutes*	0.5020	0.6267
*Chloroflexi*	0.4280	0.6172

*Stenotrophomonas* (9.2 %), *Phyllobacterium* (4.4 %), *unidentified_Cyanobacteria* (3.2 %), *Streptococcus* (3.0 %), and *Sphingomonas* (2.9 %) were the five most common genera in the human milk microbiota in the CM. In the PM, the five most common genera were *Stenotrophomonas* (8.9 %), *Pseudomonas* (8.2 %), *unidentified_Cyanobacteria* (5.3 %), *Phyllobacterium* (3.9 %) and *Lactobacillus* (3.3 %) (Fig. 1B) (Table 4).

**Table 4:** Relative OTUs percentages of the top ten genera in the human milk microbiome.

Microbiome abundance	CM (%)	PM (%)
*Stenotrophomonas*	9.2440	8.9274
*Pseudomonas*	1.3712	8.2043
*unidentified_Cyanobacteria*	3.1606	5.3248
*unidentified_Clostridiales*	2.7689	0.8930
*Phyllobacterium*	4.4248	3.8858
*Staphylococcus*	1.7610	0.1758
*Lactobacillus*	1.7672	3.3436
*Sphingomonas*	2.8781	3.1777
*Streptococcus*	3.0220	2.1532
*Bifidobacterium*	1.4588	0.5590

### 3.3 Microbiota of the infant gut at 6 months

**Fig. 2:**
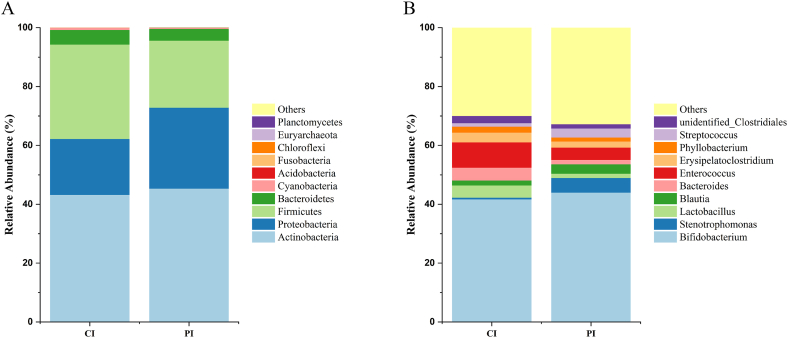
Relative abundance bar charts of the gut microbiota species. (A) Relative abundance bar charts of the top 10 phyla in the gut microbiota; (B) Relative abundance bar charts of the top 10 genera in the gut microbiota. The x-axis represents sample names; the y-axis indicates the relative abundance.

*Actinobacteria* (43.1 %), *Firmicutes* (32.0 %), and *Proteobacteria* (19.0 %) were the three most common phyla in the infant gut microbiota at 6 months in the CI. Similarly, *Actinobacteria* (45.2 %), *Proteobacteria* (27.5 %), and *Firmicutes* (22.7 %) were the three most common phyla in the PI (Fig. 2A) (Table 5).

**Table 5:** Relative OTUs percentages of the top ten phyla in the infant gut microbiota.

Microbiome abundance	CI (%)	PI (%)
*Proteobacteria*	18.9798	27.5008
*Actinobacteria*	43.0870	45.1788
*Firmicutes*	32.0205	22.7048
*Bacteroidetes*	4.9400	4.0103
*Cyanobacteria*	0.6835	0.2603
*Acidobacteria*	0.0388	0.0741
*Fusobacteria*	0.0241	0.0381
*Chloroflexi*	0.0295	0.0474
*Euryarchaeota*	0.0170	0.0004
*Planctomycetes*	0.0190	0.0194

*Bifidobacterium* (41.6 %), *Enterococcus* (8.6 %), *Bacteroides* (4.4 %), *Lactobacillus* (4.2 %), and *Erysipelatoclostridium* (3.3 %) were the five most common genera in the infant gut microbiota in the CI. In the PI, the five most common genera were *Bifidobacterium* (43.9 %), *Stenotrophomonas* (5.0 %), *Enterococcus* (4.2 %), *Blautia* (3.2 %) and Streptococcus (3.0 %) (Fig. 2B) (Table 6).

**Table 6:** Relative OTUs percentages of the top ten genera in the infant gut microbiota.

Microbiome abundance	CI (%)	PI (%)
*Bifidobacterium*	41.6348	43.8852
*Stenotrophomonas*	0.5868	5.0257
*Lactobacillus*	4.1713	1.4593
*Blautia*	1.6408	3.1628
*Bacteroides*	4.4040	1.5141
*Enterococcus*	8.5820	4.2153
*Erysipelatoclostridium*	3.3076	2.0327
*Phyllobacterium*	2.0424	1.3720
*Streptococcus*	1.1507	3.0244
*unidentified_Clostridiales*	2.4592	1.4554

### 3.4 Α-Diversity of each microbiota

#### 3.4.1 Venn graphs

**Fig. 3:**
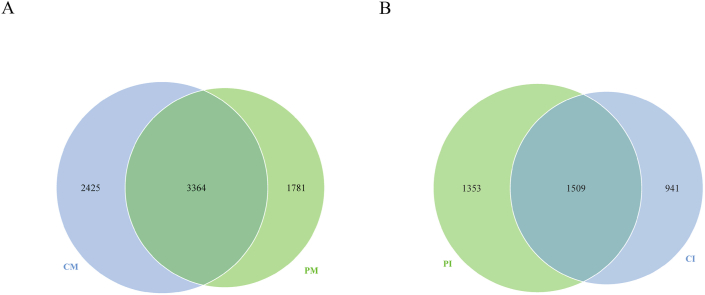
Venn diagram. A Venn diagram shows the OTUs unique to each sample and shared by different samples. Each circle represents a group, and the numbers in the overlapping regions of the circles represent the number of shared OTUs between the groups. The numbers outside the overlapping regions represent the unique number of OTUs in each group.

The OTU results were obtained by clustering. There were 3364 OTUs shared by the human milk microbiota in both the CM and the PM (Fig. 3A). The human milk microbiota in the CM had 2425 unique OTUs, while that in the PM had 1781 unique OTUs. There were 1509 OTUs shared by the gut microbiota in both the CI and PI (Fig. 3B). In total, 941 unique OTUs were identified in the gut microbiota of the CI. The PI had 1353 unique OTUs.

### 3.5 Rarefaction and rank abundance curves

**Fig. 4:**
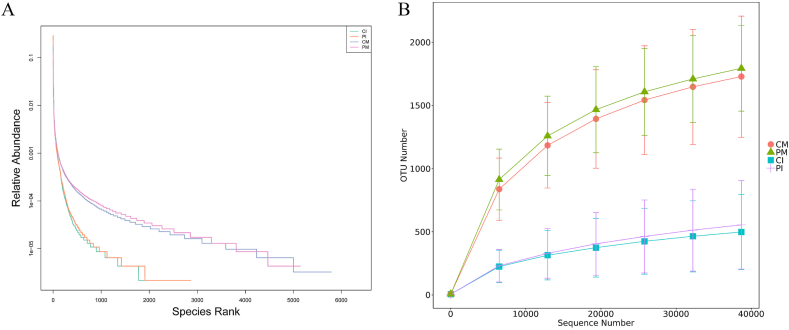
Rarefaction curve and rank abundance curve. (A) Rarefaction curve. Each curve in the diagram represents a group sample; different colors represent different groups. A flat curve indicates that a greater sequence number will lead to a lower OTU number. (B) Rank abundance curve. Each curve in the diagram represents a group sample; different colors represent different groups.

Rarefaction curves and rank abundance curves were used to analyze the α diversity of all the groups. The rarefaction curve directly reflects the rationality of the sequencing data volume; the horizontal axis represents the randomly sampled sequencing data quantity, and the vertical axis represents the observed OTU count. With increasing sequencing quantity, the trend of increase in the rarefaction curves became more gradual (Fig. 4A). In this study, the sequencing data met the sequencing requirements, and the sequencing data volume per sample was reasonable. The rank abundance curve reflects the evenness of species in the samples. In the horizontal direction, the width of the curve reflects the abundance of the species, with a wider range on the x-axis indicating greater species abundance. The shape (smoothness) of the curve reflects the evenness of the species in the sample; the smoother the curve is, the more evenly distributed the species are. The rank abundance curve showed a gradual decrease, indicating a uniform distribution of species (Fig. 4B).

#### 3.5.1 Chao1 and simpson

**Fig. 5:**
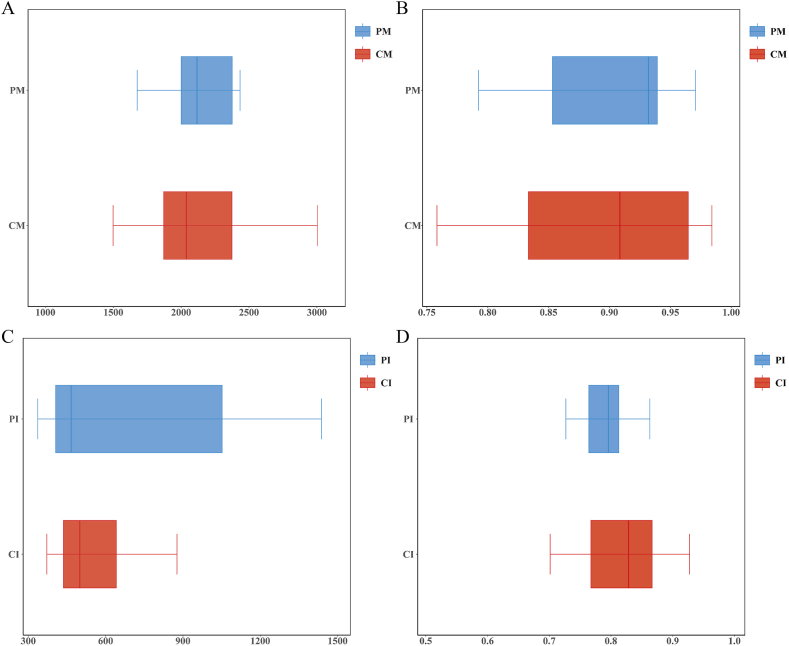
(A, C) Chao1 index and (B, D) Simpson index. The Chao1 index is used to compare the species richness of gut microbiota between different groups. A higher Chao1 index indicates greater species richness. The Simpson index is used to assess the diversity of gut microbiota between different groups. A lower Simpson index value indicates higher diversity, meaning a more even distribution of species.

The Chao1 index, which is used to estimate the number of species in the community, showed no significant differences between the CM and PM in the human milk microbiota (*P* = 0.850) (Fig. 5A). The Simpson index, which is used to evaluate the species diversity index of the microbiota, showed no significant differences between the CM and PM in terms of the human milk microbiota (*P* = 0.315) (Fig. 5B). The Chao1 index did not significantly differ between the CI and PI for the infant gut microbiota (*P* = 0.420) (Fig. 5C). The Simpson index did not significantly differ between the CI and PI for the infant gut microbiota (*P* = 0.120) (Fig. 5D).

### 3.6 NMDS analysis

**Fig. 6:**
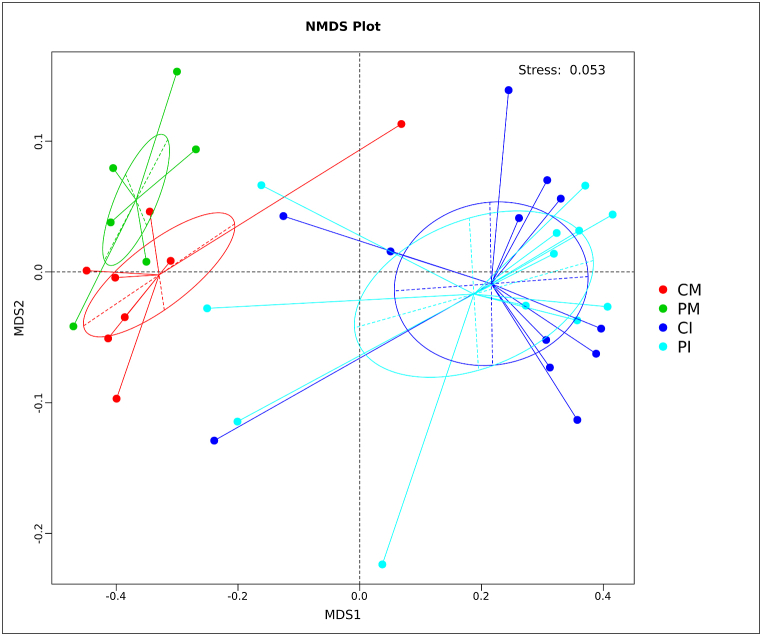
NMDS analysis of the microbiota composition. NMDS plots showing the differences in gut microbiota composition between groups. Each point represents a sample, and the distance between points reflects the similarity in microbiota composition. Stress values indicate the goodness-of-fit of the NMDS representation. Different colors represent different groups.

The NMDS analysis (stress = 0.053) indicated that the human milk microbiota compositions of the CM and PM were distinct, while the intestinal microbiota compositions of the CI and PI were similar (Fig. 6).

### 3.7 Differences between the groups at 6 months

**Fig. 7:**
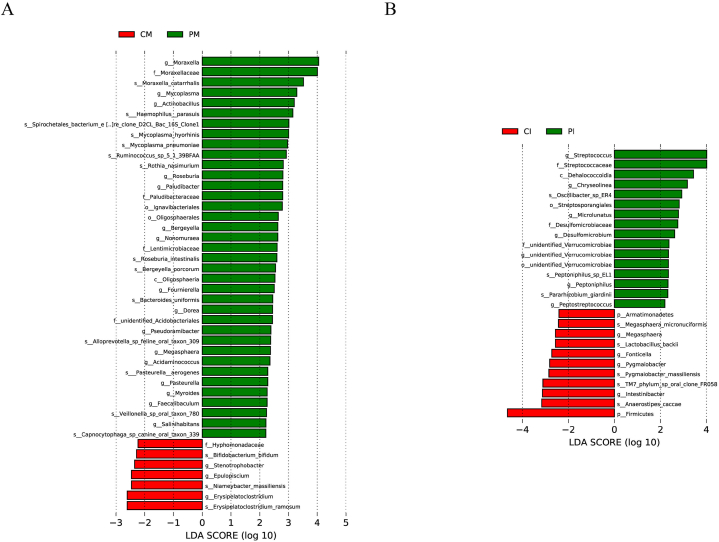
Analysis of differences in the microbiota between the two groups. (A). LDA analysis results for the bacterial milk microbiota; (B). LDA analysis results for the gut microbiota. The bar chart in the LDA score distribution displays species with LDA scores greater than the set threshold; these are biomarkers indicating statistically significant differences between groups. The length of the bars represents the magnitude of the impact of the differential species (i.e., LDA score). p__: Phylum, c__: Class, o__: Order, f__: Family, g__: Genus, s__: Species.

LEfSe was used to determine the significant differences in abundance between the different groups. As shown in Fig. 7, *Hyphomonadacea*, *Bifidobacterium_bifidum*, *Stenotrophobacter*, *Epulopiscium*, *Niameybacter_massiliensis*, *Erysipelatoclostridium and Erysipelatoclostridium_ ramosum* were significantly more abundant in the human milk microbiota of the CM than in that of the PM (P < 0.05) (Fig. 7A). *Moraxella, Moraxellaceae, Moraxella_catarrhalis, Mycoplasma, Mycoplasma_hyorhinis, Mycoplasma_pneumoniae, Bergeyella, Roseburia and Roseburia_intestinalis* were significantly more abundant in the human milk microbiota in the PM than in the CM (P < 0.05) (Fig. 7A).

*Firmicutes*, *Anaerostipess_caccae*, *Pygmaiobacter_massiliensis*, *Pygmaiobacter*, *Fonticella*, *TM7_phylum_sp_oral_clone_FR058*, *Armatimonadetes*, *Intestinibacter*, *Lactobacillus_backii*, *Megasphaera_micronuciformis*, and *Megasphaera* were significantly more abundant in the infant gut microbiota in the CI than in the PI (P < 0.05) (Fig. 7B). *Desulfomicrobium*, *Desulfomicrobiaceae*, *Oscillibacter_sp_ER4*, *Peptostreptococcus*, *Chryseolinea*, *Unidentified_Verrucomicrobiae*, *Peptoniphilus_sp_EL1*, *Peptoniphilus*, *Pararhizobium_giardinii*, *Streptosporangiales*, *Microlunatus*, *Dehalococcoidia*, *Steptococcus*, and *Streptococcaceae* were significantly more abundant in the infant gut microbiota in the PI than in the CI (P < 0.05) (Fig. 7B).

### 3.8 Human milk and infant gut microbiotas co-occurrence network analysis

**Fig. 8:**
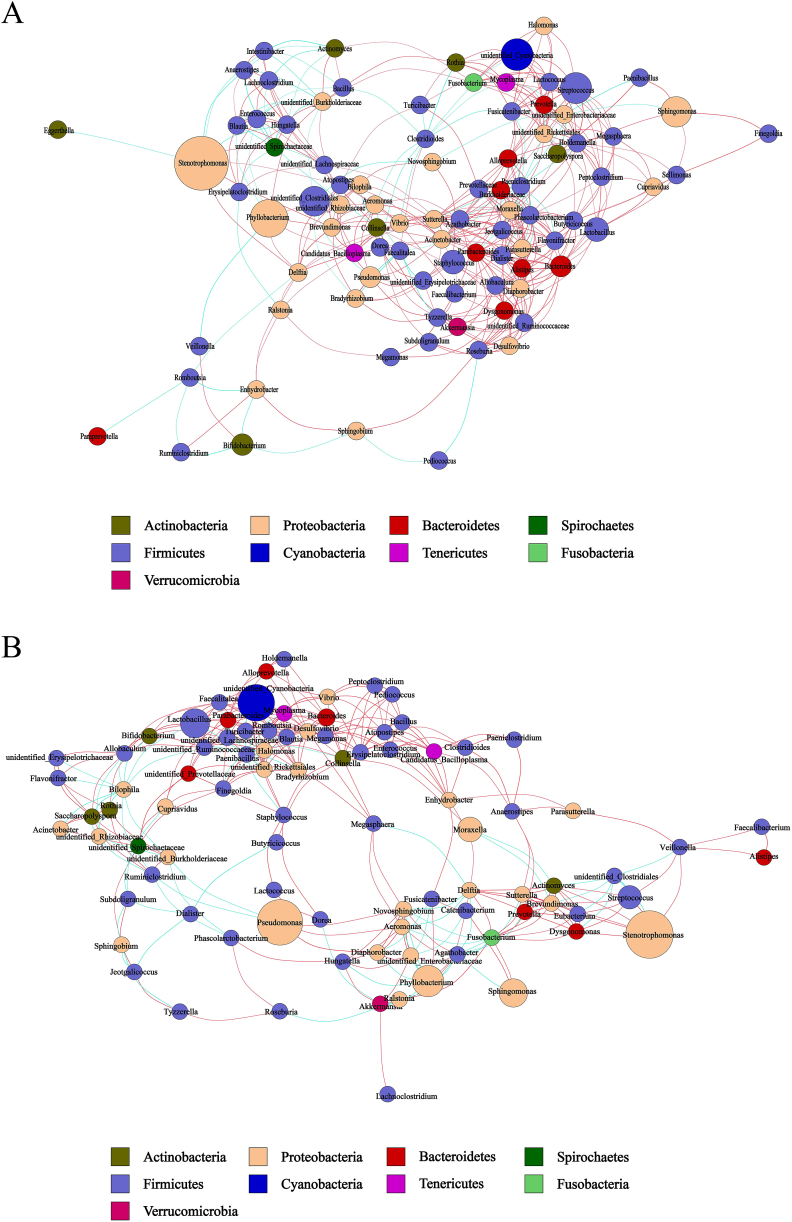
Co-occurrence networks of milk microbiota at the genus level. (A) Co-occurrence network of the bacterial milk microbiota in CM. (B) Co-occurrence network of the bacterial milk microbiota in PM. The nodes in the above figure are identified by different colors, representing each dominant genus. The line between the nodes indicates a correlation between the two genera, the red line indicates a positive correlation, the blue line indicates a negative correlation, and the thickness of the line indicates the intensity of correlation. The node size represents the content of the OTUs, and the dots of the same color represent the genus classified into the same phylum. The more connections through a node, the more associations the genus has with other members of the genus.

**Fig. 9:**
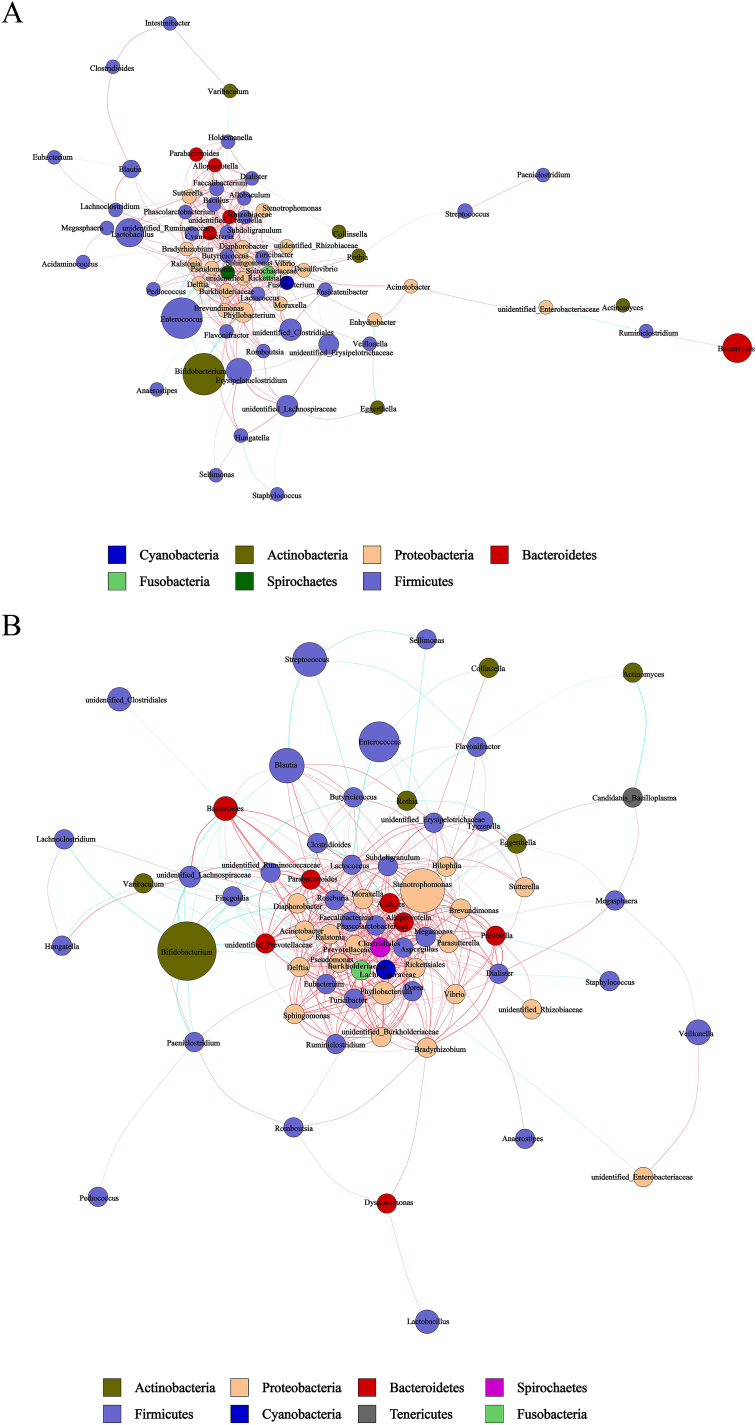
Co-occurrence networks of gut microbiota at the genus level. (A) Co-occurrence network of the infant gut microbiota in CI. (B) Co-occurrence network of the infant gut microbiota in PI. The nodes in the above figure are identified by different colors, representing each dominant genus. The line between the nodes indicates a correlation between the two genera, the red line indicates a positive correlation, the blue line indicates a negative correlation, and the thickness of the line indicates the intensity of correlation. The node size represents the content of the OTUs, and the dots of the same color represent the genus classified into the same phylum. The more connections through a node, the more associations the genus has with other members of the genus.

Microbial co-occurrence networks demonstrate the relationships of mutual influence and restriction between microbial communities (Fig. 8, Fig. 9). To further prove whether probiotic supplementation contributes to changes in the bacterial interaction network, we calculated the network index for each group (Supplemental Table 1). The network index can provide insight into the overall structure and complexity of the microbial communities, which can be influenced by probiotic intervention.

**Fig. 10:**
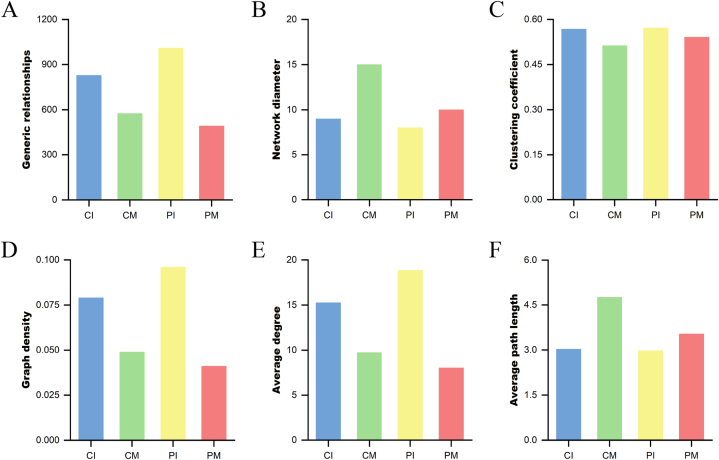
Network Index of Human Milk and Infant Gut Microbiotas. (A) Generic relationships. Shows the overall structure and connections among different microbiota species or genera. (B) Network diameter. Represents the longest shortest path between any two nodes, indicating the maximum extent of the network. (C) Clustering coefficient. Indicates the tendency of nodes to cluster together. A high coefficient shows tightly knit groups. (D) Graph density. The ratio of actual edges to the maximum possible edges, reflecting overall connectivity. (E) Average degree. The average number of connections per node, showing the level of interaction among microbiota. (F) Average path length. The average number of steps along the shortest paths between all node pairs, indicating the efficiency of interaction flow within the network.

Generic relationships refer to the number of direct interactions or edges between nodes (microorganisms) in the network. Probiotic supplementation increases the number of interactions in infant microbiotas (PI vs. CI) but decreases them in maternal milk (PM vs. CM) (Fig. 10A).

The network diameter is the longest of all the shortest paths between any two nodes in the network, providing a measure of the network's overall size and connectedness. A smaller network diameter suggests a more compact network. Probiotic groups (PI and PM) had smaller diameters compared to their control counterparts, indicating a more closely connected network (Fig. 10B).

The clustering coefficient is a measure of the degree to which nodes in a network tend to cluster together. Higher clustering coefficient indicate a more complex network with stronger interactions among microorganisms. Based on the clustering coefficients (CM: 0.513, PM: 0.541, CI: 0.568, and PI: 0.572), the clustering coefficient increased from 0.5128 in the control group to 0.5406 in the probiotic group for maternal milk, indicating an enhancement in network complexity due to probiotic supplementation (Fig. 10C). However, the impact of probiotics on the network structure of the infant microbiota was less pronounced. The clustering coefficient showed a slight increase from 0.568 in the control group to 0.572 in the probiotic group, suggesting that while probiotics do influence the infant microbiota network, the effect was not as significant as in maternal milk.

Graph density measures the proportion of potential connections in a network that are actual connections. Higher density indicates a more interconnected network. Graph density was higher in the probiotic groups, particularly in PI, indicating more interconnected networks (Fig. 10D).

The average degree measures the average number of connections each node (representing a microorganism) has in the network. In the PI, the average degree was 18.845, higher than the 15.250 in the CI, indicating more interactions per microorganism (Fig. 10E). In the PM, the average degree was 8.020, slightly lower than the 9.734 in the CM, suggesting balanced but robust interactions.

The average path length represents the average number of steps along the shortest paths for all possible pairs of nodes. In the PI, the average path length was 2.976, slightly shorter than the 3.025 in the CI, indicating more direct connections (Fig. 10F). In the PM, the average path length was 3.533, shorter than the 4.763 in the CM, suggesting a more efficiently connected network.

### 3.9 Correlation analysis between human milk microbiota and infant gut microbiota

Using Spearman correlation analysis, a heatmap was created to identify the top 20 genera of the human milk microbiota and the top 20 genera of the intestinal microbiota in 6-month-old infants, in order to explore the regulatory effects of the human milk microbiota on the infant gut microbiota.

**Fig. 11:**
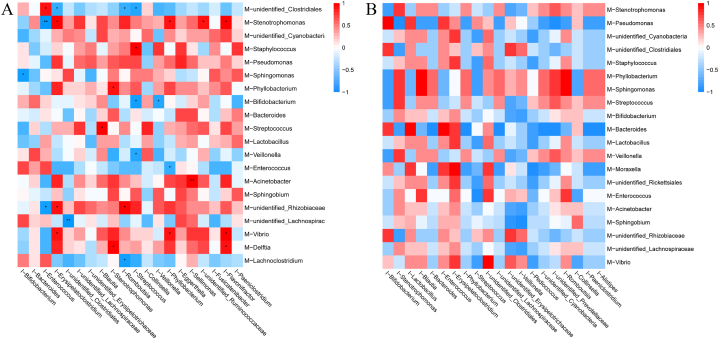
Spearman Correlation Heatmap Analysis of the Top 20 Genera Levels in the Milk Microbiome and Infant Gut Microbiome in Breastfed Infants. (A) Correlation Heatmap between CM and CI. (B) Correlation Heatmap between PM and PI. M: Human milk with bacteria at 6 months postpartum. I: Infant gut microbiotas.

The Spearman ranking for the top 20 genera between PM and PI did not find any genera showing correlation between the milk microbiome and the infant gut microbiome (Fig. 11A). However, various genera in human milk were correlated with the infant gut microbiota between CM and CI (Fig. 11B). We found that the abundance of *Bifidobacterium* in human milk was negatively correlated with the abundance of *Streptococcus* and *Veillonella* in infant feces (Table 7). The abundance of *Streptococcus* in human milk was positively correlated with the abundance of *Blautia* in infant feces. The abundance of *Sphingomonas* in human milk was positively correlated with the abundance of *Bifidobacterium* in infant feces.

**Table 7:** Correlation analysis of microbiota OTUs between the CM and the CI according to the top 20 genera (n = 7).

CM	CI	R	*p* Value
*Lachnoclostridium*	*Romboutsia*	−0.847	0.016
*Delftia*	*Stenotrophomonas*	0.847	0.016
*Delftia*	*Flavonifractor*	0.786	0.048
*Vibrio*	*Erysipelatoclostridium*	0.821	0.034
*Vibrio*	*Phyllobacterium*	0.793	0.033
*Vibrio*	*Flavonifractor*	0.893	0.012
*unidentified_Lachnospiraceae*	*unidentified_Clostridiales*	−0.964	0.003
*unidentified_Rhizobiaceae*	*Enterococcus*	−0.857	0.024
*unidentified_Rhizobiaceae*	*Erysipelatoclostridium*	0.821	0.034
*unidentified_Rhizobiaceae*	*Romboutsia*	0.857	0.024
*Acinetobacter*	*Sellimonas*	0.889	0.007
*Enterococcus*	*Phyllobacterium*	−0.77	0.041
*Veillonella*	*Streptococcus*	−0.829	0.021
*Streptococcus*	*Blautia*	0.893	0.012
*Bifidobacterium*	*Streptococcus*	−0.857	0.024
*Bifidobacterium*	*Veillonella*	−0.829	0.021
*Phyllobacterium*	*Stenotrophomonas*	0.811	0.027
*Sphingomonas*	*Bifidobacterium*	−0.857	0.024
*Staphylococcus*	*Streptococcus*	0.893	0.012
*Stenotrophomonas*	*Enterococcus*	−0.929	0.007
*Stenotrophomonas*	*Erysipelatoclostridium*	0.857	0.024
*Stenotrophomonas*	*Phyllobacterium*	0.793	0.033
*Stenotrophomonas*	*unidentified_Ruminococcaceae*	0.786	0.048
*Stenotrophomonas*	*Flavonifractor*	0.857	0.024
*unidentified_Clostridiales*	*Enterococcus*	0.829	0.021
*unidentified_Clostridiales*	*Erysipelatoclostridium*	−0.775	0.041
*unidentified_Clostridiales*	*Romboutsia*	−0.775	0.041
*unidentified_Clostridiales*	*Streptococcus*	−0.811	0.027

## 4 Discussion

Previous studies have shown that human milk is nonsterile. The presence of bacteria in sterile-collected milk samples has already been confirmed through bacterial culture methods [26]. In recent years, the development of research technologies such as Illumina sequencing has shown that highly diverse bacterial communities do exist in human milk samples through metagenomic studies [27]. The results of this experiment showed that among the 13 CM milk samples, microbiota was detected in 8 of them. In the PM samples, microbiota was detected in 6 out of 14 samples. The remaining milk samples could not be successfully sequenced, possibly due to an insufficient microbial content for optimal amplification and sequencing. In our study, *Proteobacteria*, *Firmicutes*, and *Bacteroidetes* were the three most common phyla in the human milk microbiota at 6 months in the CM. *Stenotrophomonas*, *Phyllobacterium*, *unidentified_Cyanobacteria*, *Streptococcus*, and *Sphingomonas* were the five most common genera in the human milk microbiota in the CM. The human milk microbiota in the CM group had 2425 unique OTUs. Research by Fitzstevens et al. suggested that facultative anaerobic or aerobic bacteria are the predominant colonizers of the human milk ecosystem. *Streptococcus* and *Staphylococcus* are the most common and abundant bacterial classes in human milk samples, followed by *Propionibacterium* and *Bifidobacterium*, which originate from the skin or the environment; additionally, well-known probiotic species such as *Lactobacillus* and *Bifidobacterium* can be detected in the gut [28]. Previous work showed similar results to our results, with small differences. These differences may be influenced by race, region, dietary habits, the environment and many other factors.

The intestinal microbiota of infants undergoes a dynamic and evolving process within the first year of life [[29], [30], [31]]. A study by Raspini et al. [32] revealed that the dominant phyla in the intestinal microbiota of exclusively breastfed 6-month-old infants were *Firmicutes*, *Actinobacteria* and *Proteobacteria*. In this study, at the phylum level, *Actinobacteria*, *Firmicutes*, and *Proteobacteria* were identified as the dominant bacterial groups in the CI, contrary to previous findings. At the genus level, *Bifidobacterium*, *Enterococcus*, *Bacteroides*, *Lactobacillus*, and *Erysipelatoclostridium* were the five most common genera in the infant gut microbiota in the CI group. The previous study included 6-month-old infants from Italy, and we speculate that the potential factors contributing to the differences in the infant gut microbiota could be variations in complementary feeding practices, regional disparities, and ethnic diversity.

The early establishment of the gut microbiota may directly impact future health. After birth, the intestinal microbiota of newborns is susceptible to various factors, including the mode of delivery, feeding method, gestational age, and use of antibiotics by pregnant women during pregnancy [33]. Human milk provides various important symbiotic and mutually beneficial dominant bacterial groups, such as *Bifidobacterium*, *Phyllobacterium*, *Lactobacillus*, and *Clostridium*, for the establishment of the intestinal microbiota in newborns. *Bifidobacterium*, the primary dominant colonizer, collaborates with other bacteria in the intestines to resist harmful microorganisms such as *Enterobacter* and *Salmonella*, stimulating the production of secretory immunoglobulin A (IgA) and activating T regulatory cells [34]. This collaboration promotes the development of the newborn's immune system, prevents infections, and ensures the stability of the intestinal microbiota in newborns. Human milk is the best source of nutrition for infants and is also one of the carriers of bacteria that can be transmitted from mother to baby [35,36]. In our study, we found that the infant gut microbiota at 6 months has a complicated relationship with the maternal human milk microbiota. For instance, the abundance of *Bifidobacterium* in human milk is negatively correlated with the abundance of *Streptococcus* and *Veillonella* in infant feces. The network graph showed that although Probiotic supplementation did not directly alter the abundance of probiotics, they could modify the microbial microenvironment. To further demonstrate the impact of probiotic supplementation on bacterial interaction networks, we calculated the network index for each group. This index provides insights into the structure and complexity of microbial communities [37,38]. Network index analysis reveals that probiotic supplementation significantly enhances microbial network complexity and interactions in maternal milk. This is evidenced by a smaller network diameter, indicating a more compact network, and a higher clustering coefficient, suggesting stronger node interactions [39,40]. Additionally, the network exhibits a smaller average degree and graph density, reflecting fewer interactions between nodes and less dense connectivity [41]. The shorter average path length further supports the increased complexity and robustness of microbial interactions in maternal milk due to probiotics [42]. In the infant gut microbiota, while the impact of probiotics is noticeable, it is less pronounced compared to maternal milk. The network in the infant gut microbiota shows higher graph density and average degree, indicating more interconnected and interactive nodes. It also has a shorter average path length, signifying more efficient communication between nodes. The clustering coefficient is slightly higher, suggesting somewhat stronger interactions among nodes. Overall, although probiotic supplementation does enhance microbial network complexity and interactions in the infant gut, the changes are less dramatic than in maternal milk, highlighting a more substantial impact of probiotics on the maternal milk microbiome. Not all studies have found changes in specific microbiota following probiotic supplementation. The conventional view holds that taking probiotics can increase the presence of beneficial bacteria. Research suggested that supplementing with oral probiotics during late pregnancy and the breastfeeding period can positively impact the beneficial microbiota in human milk. In a group taking VSL#3 (which includes *Lactobacillus*, *Bifidobacterium*, and *Streptococcus* thermophilus), significant increases in the amounts of *Bifidobacterium* and *Lactobacillus* were observed in both colostrum and mature milk compared to the control group [43]. However, in most cases, taking probiotics could change the corresponding microbiota, but there were some instances where it cannot be changed [44]. This relationship showed that the microbiota in human milk does not directly influence the infant gut microbiota by changing the abundance of the bacteria themselves. This influence may be reflected in variations in the infant gut environment, inducing gut microbiota variation. Our study aim was to evaluate the probiotic effect on mother milk and infant gut microbiota, as well as the correlation between mother milk and infant gut microbiota. So, we want to exclude other confounding factor to make our manuscript more rigorous. Further study is needed to explore the other factors which will influence infant gut microbiota. In addition, this study collected human milk samples from disinfected breasts, eliminating the influence of skin microbiota. However, before collecting milk, infants suckling human milk make contact with the nipple and surrounding skin. Is it possible that the combination of low CFU content in human milk and high levels of skin microbiome means that maternal skin is a more important source for infant gut colonization than human milk? The relevant mechanism of this relationship requires further investigation. The establishment of a stable gut microbiota occurs during two critical periods of transition [45]. The first transition takes place shortly after birth when newborns shift from maternal dependence to relying on human milk. The second transition happens during weaning, marked by the end of breastfeeding and the introduction of solid foods, and these changes persist until the age of three. After this point, children develop a gut microbiota structure similar to that of adults. Many studies showed that the influence of human milk microbiota, as well as the skin microbiome, was much more apparent in the early stages (2–8 weeks postpartum). However, between 3 and 6 months, particularly when early weaning begins, a significant shift in the gut microbiome occurs. This study collected human milk samples at 6 months postpartum, and infants were exclusively breastfed during this period. It might be too late to study the effect of milk microbiota on gut microbiota establishment at 6 months postpartum. The dominant genera in the infant gut can vary depending on the feeding method. In 6-month-old infants who were formula-fed, the dominant genera included *Streptococcus*, *Klebsiella*, and *Lactobacillus*. In mixed-fed infants, the dominant genera were *Streptococcus*, *Klebsiella*, and *Bifidobacterium* [46]. In this study, *Bifidobacterium* was the dominant genus in the infant gut microbiota in both the CI and PI groups, while in another similar study with exclusively breastfed infants, *Bacteroides* was the dominant genus, with *Bifidobacterium* ranking third [46]. The reason for the difference could be the variation in geographic location and environmental factors.

Previously, we investigated the impact of taking probiotics during pregnancy on the gut microbiota of pregnant women. Probiotics were taken from 32 weeks of gestation until delivery. Our study found that taking probiotics during pregnancy did not alter the alpha diversity of the gut microbiota but did affect beta diversity. Additionally, the control group showed significant depletion of *Turicibacter* and *Clostridium_sensu_stricto*, while the probiotic group experienced a significant reduction in *Phascolarctobacterium* [47]. Does supplementation with probiotics affect the establishment of milk at 6 months postpartum? In our study, at the phylum level, *Proteobacteria*, *Firmicutes*, and *Bacteroidetes* were the three most common phyla in the human milk microbiota in the PI, similar to those in the CI. The five most common genera were *Stenotrophomonas*, *Pseudomonas*, *unidentified_Cyanobacteria*, *Phyllobacterium* and *Lactobacillus* in the PI. However, the five most common genera were *Bifidobacterium*, *Stenotrophomonas*, *Enterococcus*, *Blautia* and *Streptococcus* in the CI. At the phylum level, the consumption of probiotics by pregnant women had a relatively minor impact on the composition of the infant gut microbiota at 6 months. However, at the genus level, it exerted a more significant influence on the composition of the infant gut microbiota. LEfSe was used to determine the significant differences in abundance between the different groups. The six bacteria in the human milk microbiota in the CM were significantly more abundant than those in the PM. Nine bacteria were significantly more abundant in the human milk microbiota in the PM than in the CM. According to the LEfSe results, 11 bacteria in the infant gut microbiota in the CI were significantly more abundant than those in the PI. Fourteen bacteria were significantly more abundant in the infant gut microbiota in the PI than in the CI. Probiotics cannot change the microbiota constitution directly but can change the microenvironment through their metabolites or receptor-mediated genes.

The supplementation of probiotics is a controversial topic. There have been varying opinions and research findings on whether probiotics have an impact on the structure of the microbiome. Research suggested that supplementing with oral probiotics during late pregnancy and the breastfeeding period can positively impact the beneficial microbiota in human milk. In a group taking VSL#3 (which includes *Lactobacillus*, *Bifidobacterium*, and *Streptococcus thermophilus*), significant increases in the amounts of *Bifidobacterium* and *Lactobacillus* were observed in both colostrum and mature milk compared to the control group [43]. A survey included 24 randomized controlled trials with a total of 2761 mothers and 1756 infants. The overall effect of probiotics on the detection rate of beneficial bacteria in human milk showed a risk difference of 24 % [48]. However, some research suggested that maternal probiotic supplementation did not significantly affect the overall composition of the human milk microbiota transferred during breastfeeding [[49], [50], [51]]. Our study did not find beneficial effects of probiotic supplementation on the beneficial bacteria in human milk and infant feces through analysis of LEfSe differential results. This may be related to differences in probiotic strains, dosage, administration timing, and the timing of sample collection. Further research is needed to determine the specific impact of probiotic supplementation on the abundance of beneficial bacteria in human milk and infant feces.

Our study had several limitations. We acknowledge that a major limitation of this study is the small sample size. Most ongoing studies in this area involve several hundred or even over 1000 participants to achieve sufficient statistical power for studying the high complexity of neonatal gut microbiome development. This limitation may impact the generalizability of our findings. Future studies should aim to recruit larger populations. We acknowledge the importance of a double-blind placebo-controlled design for the study's robustness. Because pregnant women are a special vulnerable group, using placebos may increase their anxiety. Therefore, we chose not to use placebos in our study. In future research, we will introduce double-blind animal experiments to better study the mechanisms involved. We recognize that another limitation of this study is the selection of a timepoint at 6 months postpartum. The influence of milk microbiota may be more direct in the earlier stages of lactation. Given that the probiotic intervention occurred during the third trimester of pregnancy, and previous studies have demonstrated that changes in the microbiota due to intervention can last several weeks before potentially reverting back to their original state, there is a question of whether any alterations in the maternal gut microbiome persist into the lactational stage, particularly up to 6 months postpartum. Spearman correlation analysis can help identify correlations between microbial communities. However, microbiome data is typically compositional, meaning each sample's total relative abundance sums to one. This constraint can lead to spurious correlations, as increases in the relative abundance of one bacterium must correspond to decreases in others, regardless of biological significance. Thus, Spearman correlations may capture spurious associations driven by these inherent constraints. For example, two unrelated bacterial species might show significant negative correlation due to the sum constraint. Additionally, microbiome data often contain many zero values, indicating that some bacteria are not detected in certain samples. Spearman correlation can be biased by these zeros, as it cannot distinguish between true absence and zeros due to detection limits. While Spearman correlation has limitations and may not always be reliable, many studies still use it to analyze relationships between microbial communities [52]. We will seek better methods in future studies to explore the relationship between human milk and the infant gut microbiota. This study lacks data on the maternal gut microbiota at 6 months postpartum, which is a key area for further investigation.

## 5 Conclusion

In this study, we demonstrated the constitution of both the human milk and infant gut microbiotas at six months postpartum. The infant gut microbiota at 6 months has a complicated relationship with the maternal human milk microbiota. At the phylum level, the consumption of probiotics by pregnant women has a relatively minor impact on the composition of the infant gut microbiota at 6 months. However, at the genus level, it exerts a more significant influence on the composition of the infant gut microbiota. However, further investigations are needed to determine the underlying mechanism involved.

### Funding

This study was supported by the Department of Science and Technology of Guangzhou (Grant no. SL2022A04J00792) and the Research Collaboration Agreement from the Third Affiliated Hospital of Sun Yat-sen University (Grant no. 202403282179), and the 10.13039/100014717National Natural Science Foundation of China (Grant no. 81771664).

## Ethics approval statement

The study project was authorized by the Institutional Review Board (IRB) for Human Subject Research at the First Affiliated Hospital of Jinan University (2019–011) and performed following the tenets of the Declaration of Helsinki. Written informed consent was obtained from every pregnant woman.

## Consent to participate

Not applicable.

## Data availability statement

All data generated or analyzed during this study are included in this published article. The sequence data reported in this study was archived in the Sequence Read Archive with the accession number PRJNA1067545.

## Code availability

Not applicable.

## CRediT authorship contribution statement

**Guangyu Ma:** Writing – review & editing, Writing – original draft, Software, Methodology. **Yimi Li:** Supervision, Investigation. **Kian Deng Tye:** Resources, Data curation. **Ting Huang:** Software, Investigation. **Xiaomei Tang:** Software, Resources. **Huijuan Luo:** Visualization, Supervision. **Dongju Wang:** Formal analysis, Conceptualization. **Juan Zhou:** Methodology, Investigation. **Zhe Li:** Writing – review & editing, Writing – original draft, Resources, Methodology, Conceptualization. **Xiaomin Xiao:** Supervision, Project administration, Investigation, Funding acquisition, Conceptualization.

## Declaration of competing interest

The authors declare the following financial interests/personal relationships which may be considered as potential competing interests:

Xiaomin Xiao reports financial support was provided by 10.13039/501100001809National Natural Science Foundation of China. If there are other authors, they declare that they have no known competing financial interests or personal relationships that could have appeared to influence the work reported in this paper.
