# Supplementary material for: The effect of oral probiotics in the last trimester on the human milk and infant gut microbiotas at six months postpartum: A randomized controlled trial
Source: Heliyon. 2024 Aug 30;10(17):e37157. doi: 10.1016/j.heliyon.2024.e37157 (PMC11402683; doi:10.1016/j.heliyon.2024.e37157)
Supplement: Multimedia component 1 [file mmc1.docx]

**Supplemental Table 1. Network Index of Human Milk and Infant Gut Microbiotas**

| **Network Index** | **CI** | **CM** | **PI** | **PM** |
| --- | --- | --- | --- | --- |
| Generic relationships | 828 | 575 | 1011 | 491 |
| Network diameter | 9 | 15 | 8 | 10 |
| Clustering coefficient | 0.568 | 0.513 | 0.572 | 0.541 |
| Graph density | 0.079 | 0.049 | 0.096 | 0.041 |
| Average degree | 15.250 | 9.734 | 18.845 | 8.020 |
| Average path length | 3.025 | 4.763 | 2.976 | 3.533 |
